# Mantle upwelling beneath the Apennines identified by receiver function imaging

**DOI:** 10.1038/s41598-020-76515-2

**Published:** 2020-11-12

**Authors:** Claudio Chiarabba, Irene Bianchi, Pasquale De Gori, Nicola Piana Agostinetti

**Affiliations:** 1 Istituto Nazionale di Geofisica e Vulcanologia, Rome, Italy; 2 Department of Meteorology and Geophysics, University of Vienna, Vienna, Austria; 3 Department of Earth and Environmental Sciences, Universita′ di Milano Bicocca, Milano, Italy

**Keywords:** Geodynamics, Geophysics, Seismology, Tectonics

## Abstract

Magmatism, uplift and extension diffusely take place along collisional belts. Even though links between mantle dynamics and shallow deformation are becoming more evident, there is still poor understanding of how deep and surface processes are connected. In this work, we present new observations on the structure of the uppermost mantle beneath the Apennines belt. Receiver functions and seismic tomography consistently define a broad zone in the shallow mantle beneath the mountain belt where the shear wave velocities are lower than about 5% and the Vp/Vs ratio is higher than 3% than the reference values for these depths. We interpret these anomalies as a pronounced mantle upwelling with accumulation of melts at the crust-mantle interface, on top of which extensional seismicity responds to the crustal bending. The melted region extends from the Tyrrhenian side to the central part of the belt, with upraise of fluids within the crust favored by the current extension concentrated in the Apennines mountain range. More in general, mantle upwelling, following detachment of continental lithosphere, is a likely cause for elevated topography, magmatism and extension in post-collisional belts.

## Introduction

**Figure 1:**
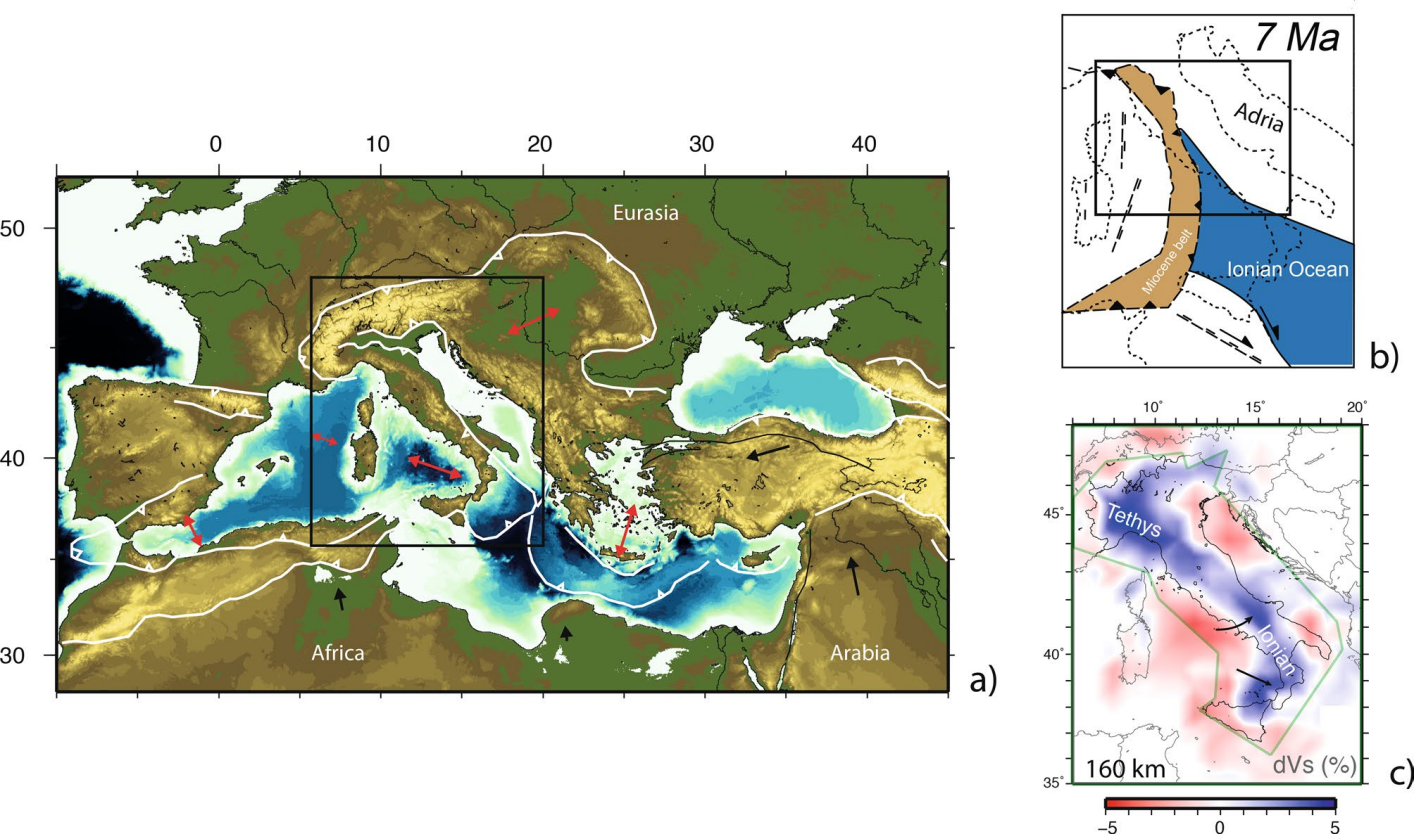
Schematic map of the Mediterranean region (**a**), showing the main compressional fronts of the broad Alpine belt (white lines), the direction of stretching of back-arc basins (red arrows), and the general direction of major plates (with respect to a fixed Eurasia). Sketch of the central Mediterranean area (**b**) at 7 Myr, modified from Magni et al.^18^, Vs model showing traces of the high velocity Ionian slab dived in the upper mantle (**c**) from Giacomuzzi et al.^19^.

Collision of buoyant continental terranes occurs usually at the end of oceanic subduction^1^. Under-thrusting of continental crust and lithosphere produces mountains whose origin is related to processes that include isostatic rebound, convective instability of thickened lithosphere, and stress generated by mantle convection^2–5^. The Alpine mobile belt (Fig. 1a) is a prime example of deformation over horizontal distances that are much larger than plate thickness and without sharp plate boundaries^6^. The central segment of the mobile belt has been shaped since the Cenozoic by the subduction of the Tethys and Neo-Tethys oceans and the collision of several continental slivers embedded between the oceanic lithosphere (Fig. 1b^7,8^). Kinematic models for the evolution of this system have been proposed based on tomographic imaging^9–13^, in which the small-scale anomalies have been interpreted in terms of volcanism, indicating slab tearing or detachment and sub-lithospheric mantle replacement after delamination^14–17^.

The Apennines are a key place for exploring how extension develops over a compressional belt, thanks to the vast abundance of geologic, seismologic and geodetic data^1,7,10,11,20–22^. The Quaternary extension replaced Pliocene compression, with volcanism spreading on the internal Tyrrhenian sector^23–25^. Plio-Quaternary volcanism features strong compositional variations, suggesting complex mantle sources, with mixed subduction-related and intraplate signatures^26^. Although the imprint from subducted material is evidenced, the way in which magmatism and extension are entangled with the continental subduction of Adria still remains unclear.

The large set of high-quality seismological data recorded by broad-band stations has supported several studies on the crust and mantle structure of the area. Tomographic images show the remains of the subducted lithosphere in the upper mantle beneath the Apennines^9,12,13,19,27^ (see Fig. 1c). Anyway, the resolution of the published models is still not adequate to infer how tectonic processes are linked to surface geology, magmatism and seismicity. Interpretation based on such models can in fact support different evolutionary models for the area, ranging from still active subduction to collision and delamination. Receiver functions (RFs) studies were done to map the Moho and LAB depth^28–30^, address the style of tectonics evolution of the belt^31^, outline fluid migration from dehydrating subducted lithosphere^32^ and define geometry of subducting lithosphere^33^. In particular, a diachronous and heterogeneous (in space) process of lithospheric underplating versus delamination-retreat has been observed in the Apennines, while broad asthenosphere upwelling postulated in slab retreat models seems to be absent^34^. Such findings support the idea that part of the Quaternary volcanism and the uplift are associated with lithospheric delamination^35^ and mantle dynamics^36^.

**Figure 2:**
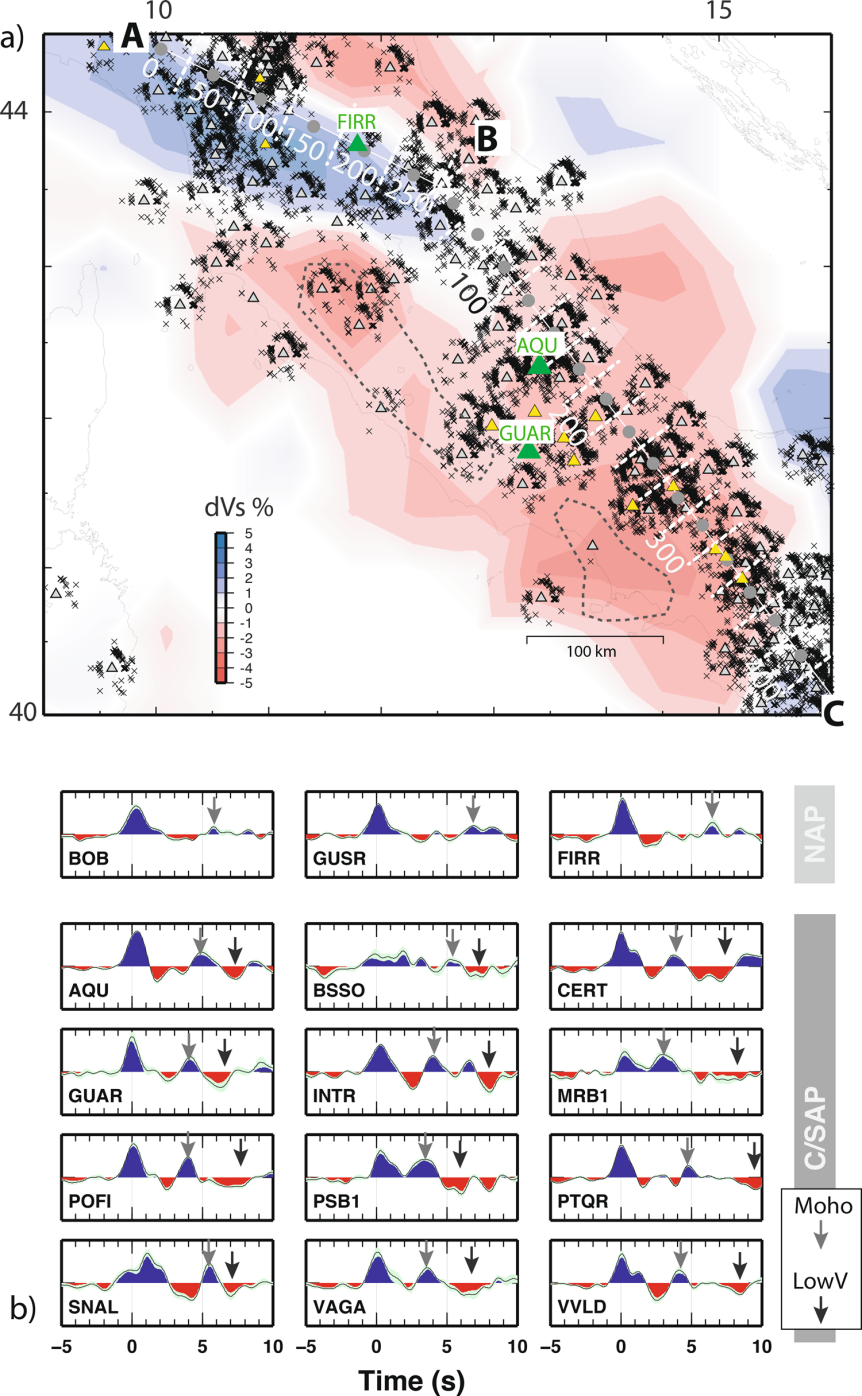
(**a**) Broad band seismic stations (triangles) and piercing points (gray crosses) at 70 km depth of the teleseismic events used for the RF’s dataset, background colors show the Vs anomalies of the uppermost mantle in a layer at 60 km depth (from^19^). The white lines are the traces of the profiles shown in Fig. 4 (the dashed lines mark the area over which the RFs are used for the stacking of each RF gather), gray circles highlight the “spots” with a 50 km spacing for the AB profile and with 25 km spacing for the BC profile. Green triangles are the stations for which the 1D Vs model is computed (see Fig. 3). (**b**) The isotropic component (k = 0) of the RF for selected permanent seismic stations (yellow triangles in the figure), showing the main pulses of the lithosphere. Gray and black arrows indicate the Moho discontinuity and the negative pulses in the upper mantle. The location of the stations is shown in Fig. SOM1. The Quaternary volcanic areas are shown by black dashed lines.

The aim of this study is to focus on this aspect, trying to elucidate structural clues for mantle dynamics. To do so, we model a vast set of RFs from temporary and permanent broadband stations to focus on the structure of the uppermost mantle along the entire Apennines belt (Fig. 2a). RFs have been stacked and depth migrated to image the shear-wave velocity at the top of the mantle on a profile running along the entire Apennines belt. For three representative stations of the main Apennines domains, we have computed a 1D Vs model following a trans-dimensional Monte Carlo inversion of RF data^37^.

**Figure 3:**
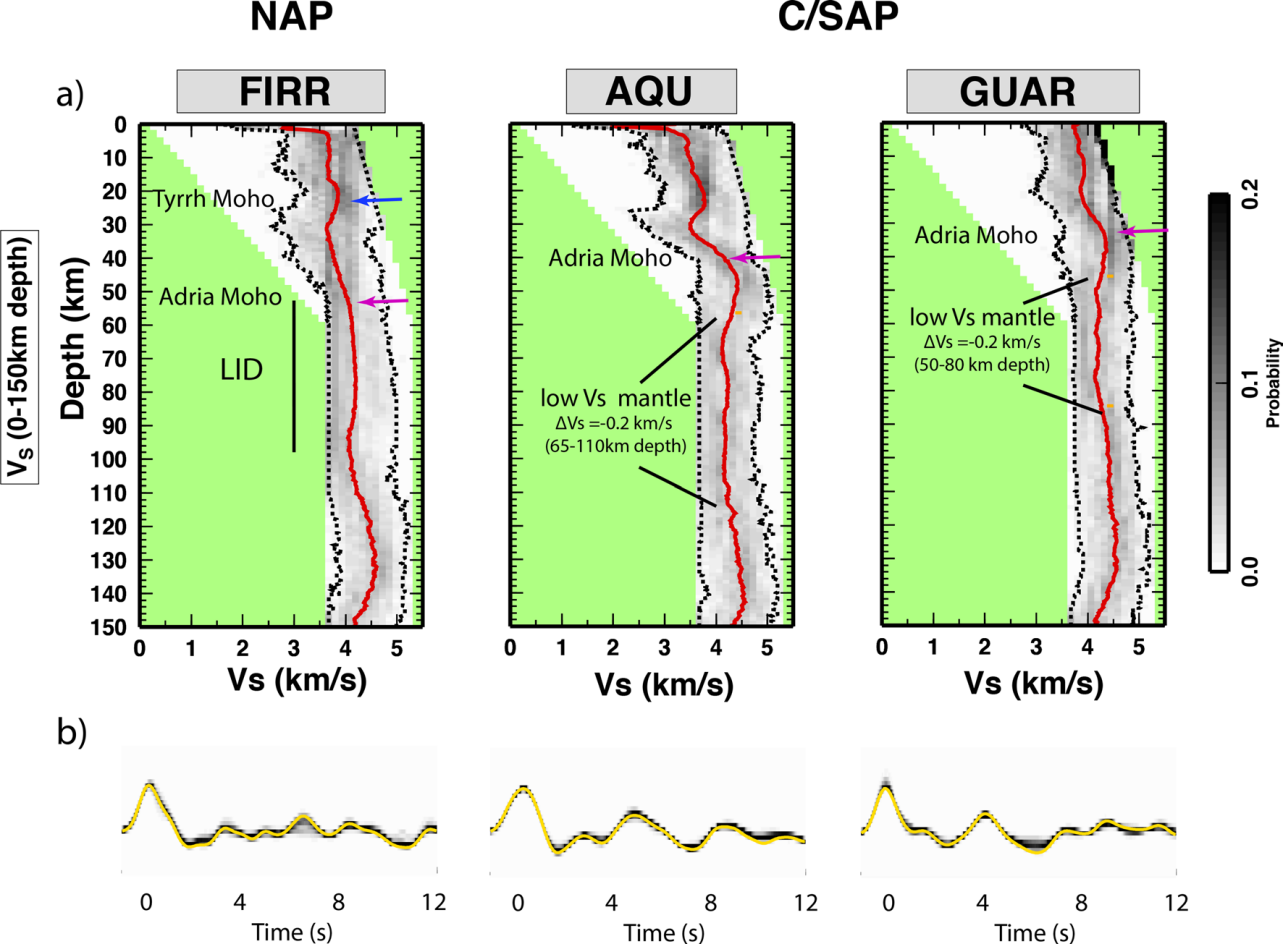
1D velocity profiles at FIRR, AQU and GUAR representative for the NAP and C/SAP domains. (**a**) Vs models: the posterior probability distribution of Vs down to 150 km depth, with the mean value (red line) and 2-s confidence interval (dashed lines). The Tyrrhenian and Adriatic Moho are indicated by blue and purple arrows respectively, while the orange dashed line and the orange arrows point to the low Vs and high Vp/Vs in the mantle. (**b**) Observed (yellow) versus synthetic k = 0 harmonic of the RF. Note the difference between the two domains in the top of the Adria mantle (60–90 km depth) and the strong correspondence with indications from tomography.

Finally, we interpret our new results jointly with the high-resolution Vs and Vp/Vs tomographic models obtained by Giacomuzzi et al.^19^, hereafter called G12. The agreement between shear wave velocities from these tomograms and the depth of low velocity layering in mantle enables a robust interpretation of the uppermost mantle structure, enlightening active processes underneath the belt.

## Results

We compute RF from teleseismic events by suing a Gaussian filter with a cut-off frequency of about 1 Hz to emphasize the features within the upper 150 km depth. We apply angular harmonic decomposition on the RF data-set to isolate the constant (or k = 0) contribution, i.e. the amplitudes in the RF time-series which is related to converted phases generated at velocity discontinuities between isotropic layers (see “Data and methods” section). First, we show and give preliminary interpretation to the constant component (k = 0) of the RF, as representative example of the heterogeneity in the mantle structure of the Apennines (Fig. 2b). RFs in the Northern (NAP) and the Central-Southern Apennines (C/SAP) show different pulses related to main discontinuities in the crust and upper mantle. In the C/SAP, we observe a clear positive phase generated at the Moho interface (marked by a gray arrow in Fig. 2b), which arrival time after the P-direct is between 3 s (e.g. MRB1) and 5.5 s (e.g. SNAL). A later negative phase caused by a decrease in acoustic impedance (marked with black arrow in Fig. 2b) is found with arrival time between 6 s (e.g. GUAR) and 9 s (e.g. PTQR), with an amplitude larger than amplitudes on the pre-signal window. This phase corresponds to a decrease of velocity in the upper mantle. Mantle-related negative pulses in the k = 0 are a persistent feature of the RF at stations located in C/SAP. Some of the C/SAP stations show a positive phase between the direct P and the Moho pulse. These stations are located on the mountain chain, and the signal cannot be due conversions at the interface of shallow sedimentary cover, rather it is due to a mid-crustal discontinuity already investigated^38^. In the NAP instead, we notice the lack of such distinct negative phase, while we observe a main positive pulse at arrival times between 6 and 7 s (stations BOB, GUSR and FIRR between 8 and 9 s delay time in Figs. 2, 3). These features signify the presence of a deep interface, with positive impedance contrast below the Moho, which is also consistent with previous findings by Bianchi et al.^33^ and Chiarabba et al.^34^.

Considering the whole area covered by our study, we can now infer the presence of significant lateral heterogeneity in the mantle of the Apennines belt, with positive and negative Vs at around 60 km depth in the northern (NAP) to the central/southern (C/SAP) sections of the mountain chain, respectively.

### 1D Vs profiles

In order to achieve quantitative support to the RFs migrated stack and the comparison between RFs and tomographic models, we compute 1D velocity models for three stations that are representative of the two NAP and C/SAP domains (Fig. 3) and have a complete set of RFs covering the entire range of back-azimuth. RF inversion is performed using a trans-dimensional Markov chain Monte Carlo sampling of the parameter space^37^, which allows to obtain a data-driven resolution in the final model and to retrieve statistically significant features in the Vs structures. Station FIRR is located in the NAP domain; the mean model, that we use here for interpreting the PPD, shows three main features: an increase in velocity at about 25 km depth that we interpret as due to the Tyrrhenian Moho, and another increase in velocity at about 55 km depth, that we interpret as the Adriatic Moho. Below (between 60 and 100 km depth) we interpret the stable velocity plateau as the Adriatic LID. Stations AQU and GUAR instead are located in the Central Apennines; both of them show one major positive velocity step at depth (at about 40 and 35 km depth respectively) followed by a decrease in Vs (4–5% reduction, ∆Vs =  − 0.2 km/s with respect to the AK135 velocity model) down to 120 and 90 km respectively, where *V*_*s*_ increases again.

In particular, we highlight that a Moho doubling (Tyrrhenian, shallower, and Adriatic, deeper, Moho) is well defined at station FIRR in NAP, where also the uppermost mantle has consistent high Vs, in agreement with previous studies (e.g.^32,39^). Instead, a Moho doubling is not observed in C/SAP (stations AQU and GUAR), and here the uppermost mantle underneath the Adria Moho has a Vs reduction, in agreement with the results presented in Di Bona et al.^40^.

### Vs and Vp/Vs model of the uppermost mantle along the Apennines belt

**Figure 4:**
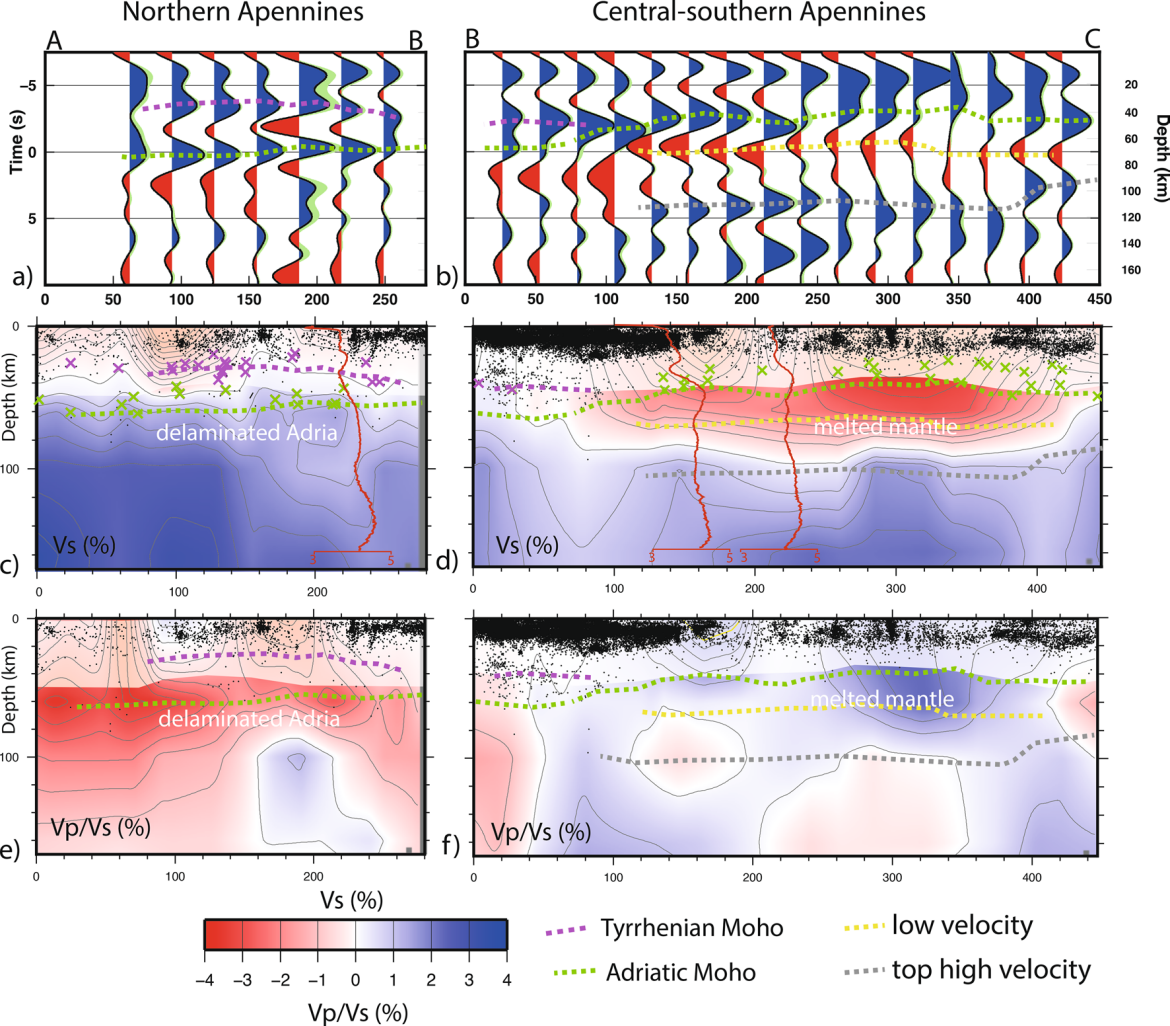
Velocity profile of the Vs structure as defined by RF stacking for the northern (**a**) and central-southern Apennines (**b**). Dashed lines are: Adria Moho (green), Tyrrhenian Moho (purple), upmost mantle low Vs (yellow), high Vs detached lithosphere (gray). Velocity profiles of Vs (**c**,**d**) and Vp/Vs (**e**,**f**) from the G12 model (with masking of the shallow poorly resolved features). Seismicity occurring at ± 30 km from the line are taken from Chiarabba et al.^41^. Crosses are the Moho depth from Piana Agostinetti and Amato^30^ and Piana Agostinetti^39^, green = Tyrrhenian neo-formed Moho, purple = Adria Moho. The mean 1D Vs profiles for the three stations are plotted on the tomographic Vs model. Note the decrease in seismicity above the top of the low Vs anomaly.

To enhance the structural difference of the mantle structure, we draw a transect along the Apennine belt high topography (AB for NAP, BC for C/SAP, located north and south of latitude 43° N, see Fig. 4). Information about the crustal and mantle structures can be derived by jointly interpreting the RF profiles (a,b panels) with tomographic slices (c–f) derived from the G12 model (see Fig. SOM5).

The stacked RF along profiles evidences a clear continuous Moho signal that splits into two distinct pulses in the NAP, differentiating the Tyrrhenian (shallower) and the Adriatic (deeper) Moho. These features are consistent with RF modeling (Fig. 3) and Moho estimates obtained with different methods^30,39,42,43^.

The heterogeneity in the Vs structure along the belt is clearly visible in the shear-velocity structure independently resolved by the two methods. In the NAP sector, a clear positive pulse is centered at around 70 km depth consistent with high velocity anomaly (Fig. 4a,c), while negative pulses are evident at the same depth in the C-SAP sector and correspond to a large low Vs anomaly in tomograms (Fig. 4b,d). The observed remarkable consistency between the positive and negative pulses of the RF, the 1D Vs profiles, and the tomographic Vs model give confidence in the reliability of this velocity pattern.

## Discussion

Extension along the Mediterranean-Alpine belt started in several locations at different times, creating rifting systems and back-arc basins^18,44,45^ and spreading over wide continental areas like the Pannonian basin, the southern Tyrrhenian and Apennines, central Greece and Anatolia^46–48^. Extension is widely documented by GPS data, although with relative velocities that change also across short distances^49–51^.

The dynamics of the Mediterranean system is thought to be primarily governed by subduction, with small scale convection that re-shapes the plate boundary configuration^10,11^. In the central Mediterranean area, the heterogeneous evolution of Ionian subduction since about 35 Ma^10,11,19,21^ and the interaction with continental margins, creates changes in tectonics and surface expression at a scale of tens-to-hundreds of kilometers.

We present new insight into the mantle structure in the central segment of the mobile belt, where belt-perpendicular extension and uplift replaced compression since about 1 Ma. The remarkable agreement between negative and positive pulses in migrated RFs and tomographic anomalies gives a very robust indication about the structure and physical state of the uppermost mantle (Fig. 4).

Positive pulses and high Vs coherently demark the Adria continental lithosphere delaminated and detached under the northern sector of the belt, while negative pulses and low Vs extend in the uppermost part of the mantle beneath the high topography portion of the central/southern Apennines belt.

Beneath NAP, the Adria continental lithosphere is delaminated with sub-lithospheric mantle substitution underneath the young and shallow Tyrrhenian Moho. This process is well documented by the peeled-off lithosphere defined by RFs transects across the belt (see^33,34^), which remnant here is evidenced by the positive pulses and high Vs features at depth higher than 60 km (X = 50–250, Fig. 4c).

In the central-southern Apennines, we observe negative pulses at around 60–70 km depth, consistent with the top of a diffuse zone of low *Vs* and high *Vp/Vs* (Figs. 4b,d,f). The remarkable coincidence between low *Vs* and strongly positive *Vp/Vs* is coherent with either a strongly hydrated upper mantle or diffuse partial melting. In the former case, the anomalous mantle can derive from previous tectonics events (past subductions or continental dismembering during the Mesozoic rifting). Anyway, we are attracted by the explanation in terms of melted mantle, also because the western side of the area experienced intense magmatism since about 4–5 Myr, prolonged until present^25^. Negative perturbations like those observed here are consistent with a 0.5–1.0% of partial melting in the mantle^52,53^. This broad anomaly is bottomed by positive pulses and high velocity coherent with deep detached lithosphere, in agreement with broad scale tomographic models (e.g.^13^).

We hypothesize a large episode of mantle upwelling beneath the central-southern Apennines, that followed the detachment of the subducted oceanic lithosphere, actually imaged in the mantle at depth below 100 km^19,27^. Similar zones with diffuse partially molten zone near the crust–mantle transition have been imaged beneath the Puna cordillera system and quoted to generate mid-crustal magmatic bodies that could trigger igneous activity and volcanism (see MASH in^54^). The creation of such large lithospheric-scale magma plumbing system is favored by the lower densities of the crustal material at the interface that force the denser mantle-derived melts to stall and differentiate. The crustal belt-perpendicular extension of the Apennines can be partly supplied by melts upraise within the crust, or alternatively may condition the local upraise. Similar accumulation of melts at the crust-mantle transition may account for extension and magmatism in continental areas. Seismicity follows the general pattern of the upwelling mantle, with crustal extension roofing on top of the upwelling zone, locally rarefied in correspondence of the locked segments of the normal faulting system (Fig. 4). Deeper sub-crustal seismicity is restricted to the northern portion of the belt, where continental delamination still proceeds^34^.

The high temperature at the crust-mantle transition sustained by the melt portions can be the cause of the observed uplift and extensive degassing^35,55^ and Quaternary magmatism in the western, stretched portion of the belt^24^. Our results support the hypothesis that the elevated topography and crustal extension along the Apennine belt are driven by stress generated at the base of the lithosphere (e.g. see^36^), by mantle upwelling that follows the detachment of the slab. Seismicity follows the crustal bending on top of the upwelling mantle, creating the diffuse extension within the Adria lithosphere. Independent geophysical methods recovered such complexity in the upper mantle of the Apennines, thanks to the abundant high quality dataset. Our results can be generalized to other cases of post-collisional extension and magmatism spreading over the Alpine belt and development of a Cordilleran margin in mountain belts.

## Conclusions

Teleseismic RFs and tomography consistently reveal broad low *Vs* and high *Vp/Vs* anomalies in the uppermost mantle beneath the Apennines. We interpret such anomalies as evidence for diffuse mantle upwelling and accumulation of melts at the crust- mantle transition, which lead the belt-perpendicular extension and uplift in the last 1 Ma. While the mantle upwelling is the cause of the current extension, melts accumulation accounts for the Quaternary volcanism that until now pervaded the stretched Tyrrhenian side of the area. The peculiar signature of mantle melts is likely related to the previous subduction process. 

This mechanism can be potentially applied to other cases of extension that spread over wide continental regions. The low Vs melt zone is not restricted to the mantle beneath the Quaternary volcanic areas but is located under the Apennines suggesting future broad effects on a large scale, as soon as the extension will favor the upraise of fluids within the crust. In our hypothesis, local batches of melts can reside in the lower crust of the stretching area.

## Data and methods

We compute RFs from a new database of three-component broadband records of teleseisms recorded at a total of 105 temporary and permanent seismic stations operating in the central Mediterranean area during the past decade (Fig. SOM1), and show them on profiles running along the Apennines belt (data in https://www.orfeus-eu.org/data/eida). From 20 K teleseismic events that occurred at an epicentral distance between 30° and 100°, with magnitude Mb > 5.5, 6500 high-quality RFs were obtained. These were generated by frequency domain deconvolution of the vertical component of the seismic records from their radial components^56^, following the approach developed by Di Bona et al.^57^. To focus on the main lithospheric structures, we employ a Gaussian filter with a = 2 (see^57^), i.e. with a cut-off frequency of about 1 Hz to emphasize the features within the upper 150 km depth. We apply angular harmonic decomposition on the RF data-set to isolate the constant (or k = 0) contribution, i.e. the amplitudes in the RF time-series which are independent of the back-azimuth of the incoming P-wave^58^. This constant component is related to converted phases generated at velocity discontinuities between isotropic layers, while the higher-order harmonics (i.e. k = 1, 2) retain the amplitudes of those converted phases due to heterogeneities such as dipping discontinuities or anisotropic layers (e.g. see^33,59^). Employing the harmonic decomposition of the RF data-sets has been proven to be advantageous with respect to the simple analysis of stacked RF, due to the capability of isolating the isotropic (in the k = 0) from non-isotropic (in k = 1, 2) phases. In order to improve imaging of the uppermost mantle just underneath the Moho**,** RF are migrated at 70 km depth (using the AK135 velocity model^60^), and stacked along profiles by using a common-conversion-point (CCP) approach^33,61,62^. The CCP approach guarantees the suppression of multiple phases and retains coherent phases between nearby stations. The profiles have been sampled assuming central spots with a distance of 25 km. RF from different stations are associated to a spot if their piercing point at 70 km depth is located in a 25 × 50 km box centered on the spot itself (Fig. SOM 1). Angular harmonic decomposition is applied to the RF gathered at each location; in this work we describe the k = 0 component of the harmonic decomposition (Fig. SOM2) as representative of the 1D structures below the Apennines.

For testing the reliability of this first order difference, we compute synthetic RFs for three velocity models (with high or low Vs in the upper mantle) to be compared with the observed wiggles representative of the two domains (Fig. SOM3). The synthetic RFs have been computed by using RAYSUM (Frederiksen and Bostock^63^), which is a theoretical method that is used to model the teleseismic waves at a given recording site; the velocity models for the recording sites are shown in Fig. SOM3b,e,g, and represent the interpreted structures. For the NAP the Tyrrhenian Moho is located at 30 km, and the top of delaminated Adria is at 60 km depth, both are marked by an increase in velocity. We compare this synthetic wiggle with the observed wiggle at 125 km along profile AB, which summarizes the major features we describe and interpret here. We see that two main interfaces associated with increases in Vs generate the two observed positive phases. The two interfaces are schematically representative of the Tyrrhenian and Adriatic Moho (at 30 and 60 km depth, respectively) in the area of crustal thickening and doubling. For C/SAP, we test two velocity models (shown in Fig. SOM3e,g). Both models have the same velocity increase at 45 km depth, representing the Adriatic Moho, but they differ at depth between 60 and 90 km. The first model (Fig. SOM3e) has a velocity decrease of about 8% in this depth interval, representing a partially melted upper mantle; the second has a constant velocity below the Moho (Fig. SOM3g). Synthetic RFs obtained by the first model show a clear negative pulse fitting the observed data (Fig. SOM3d), and clearly supporting the existence of a pronounced velocity reduction in the uppermost mantle.

We also compute a 1‐D Vs profile for three seismic stations representative of the structural difference between the NAP and C/SAP domains (see Fig. SOM1), following a trans-dimensional Monte Carlo inversion of RF data^37^. The k = 0 harmonics and their standard deviation are used to retrieve the isotropic structure of the lithosphere, by computing the posterior probability distribution (PPD) of S-wave velocity at depth with a reversible jump Markov chain Monte Carlo approach (RjMcMC^37^). The a priori probability distributions of *S-wave* velocity and *V*_*p*_/*V*_*s*_ are considered Gaussian. For S-wave velocity, we used a depth-dependent prior distribution with a mean Vs increasing from 2.2 km/s at the top to 4.45 km/s at 60 km depth of the model. In the same depth interval, prior standard deviation decreases from 0.5 to 0.2 km/s. Deeper than 60 km, the prior distribution is constant with a mean value of 4.45 km/s and a standard deviation of 0.2 km/s. The prior mean value of *V*_*p*_/*V*_*s*_ was set to 1.75 with 0.05 of standard deviation, regardless of the depth-level. Conversely, we used uniform prior distributions for the number of interfaces, and for the depth of the interfaces. The number of interfaces in the models is uniformly distributed between 1 and 100, while their minimum and maximum depths are 0 and 150 km, respectively (maximum length of Radial Receiver Function, RRF = 40 s). The likelihood function between observed and synthetic RFs is estimated using a full covariance matrix for errors^37^. The RjMcMC algorithm is used to sample 400,000 models for each Markov chain, for an ensemble of 40 million velocity models by running 100 RjMcMC parallel Markov chains for each station. The output of the RjMcMC sampling gives the PPD of the *V*_*s*_ and of the *V*_*p*_*/V*_*s*_, the mean model, and the distribution of seismic interfaces at depth (Fig. SOM4).

## Supplementary information

Supplementary Information.
