# Supplementary material for: Mantle upwelling beneath the Apennines identified by receiver function imaging
Source: Sci Rep. 2020 Nov 12;10:19760. doi: 10.1038/s41598-020-76515-2 (PMC7661539; doi:10.1038/s41598-020-76515-2)
Supplement: Supplementary file 1 — Supplementary Information. [file 41598_2020_76515_MOESM1_ESM.pdf]

Supplementary material for paper:

Mantle upwelling beneath the Apennines identified by Receiver Function imaging

Claudio Chiarabba[1], Irene Bianchi[1,2,], Pasquale De Gori[1,], and Nicola Piana Agostinetti[1,3]

1 Istituto Nazionale di Geofisica e Vulcanologia, Rome, Italy

2 Department of Meteorology and Geophysics University of Vienna, Vienna, Austria

3 Department of Geodynamics and Sedimentology, University of Vienna, Vienna, Austria

Corresponding Author: claudio.chiarabba@ingv.it

The supporting material consists of five figures showing station locations (SOM1), RF data (SOM2), synthetic test for consistency of structural patterns (SOM3), full details of 1D velocity profiles (SOM4) and tomographic layer of Vs and Vp/Vs at 60 km depth (SOM5).

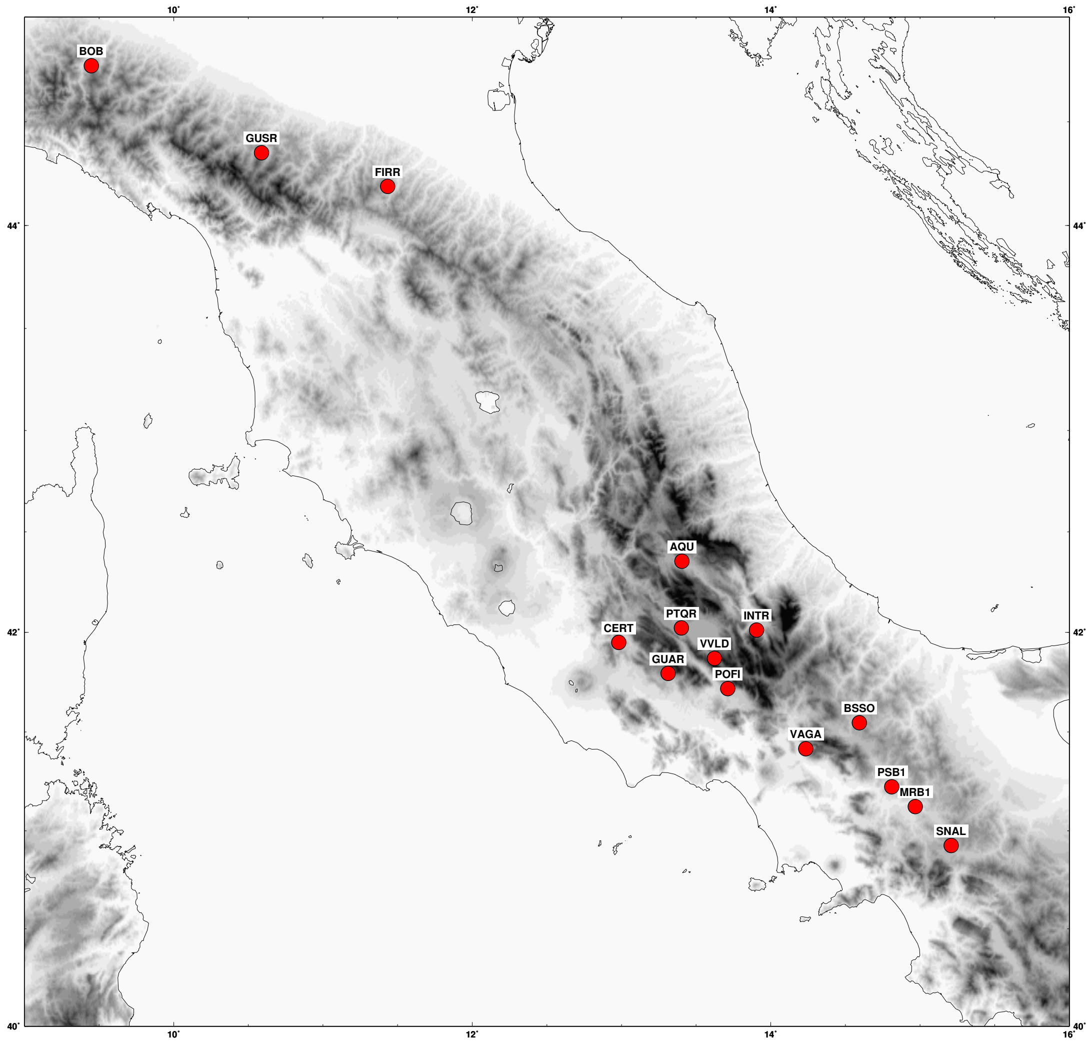

Figure SOM1: map of the seismic stations which data are shown in figures 2 and 3.

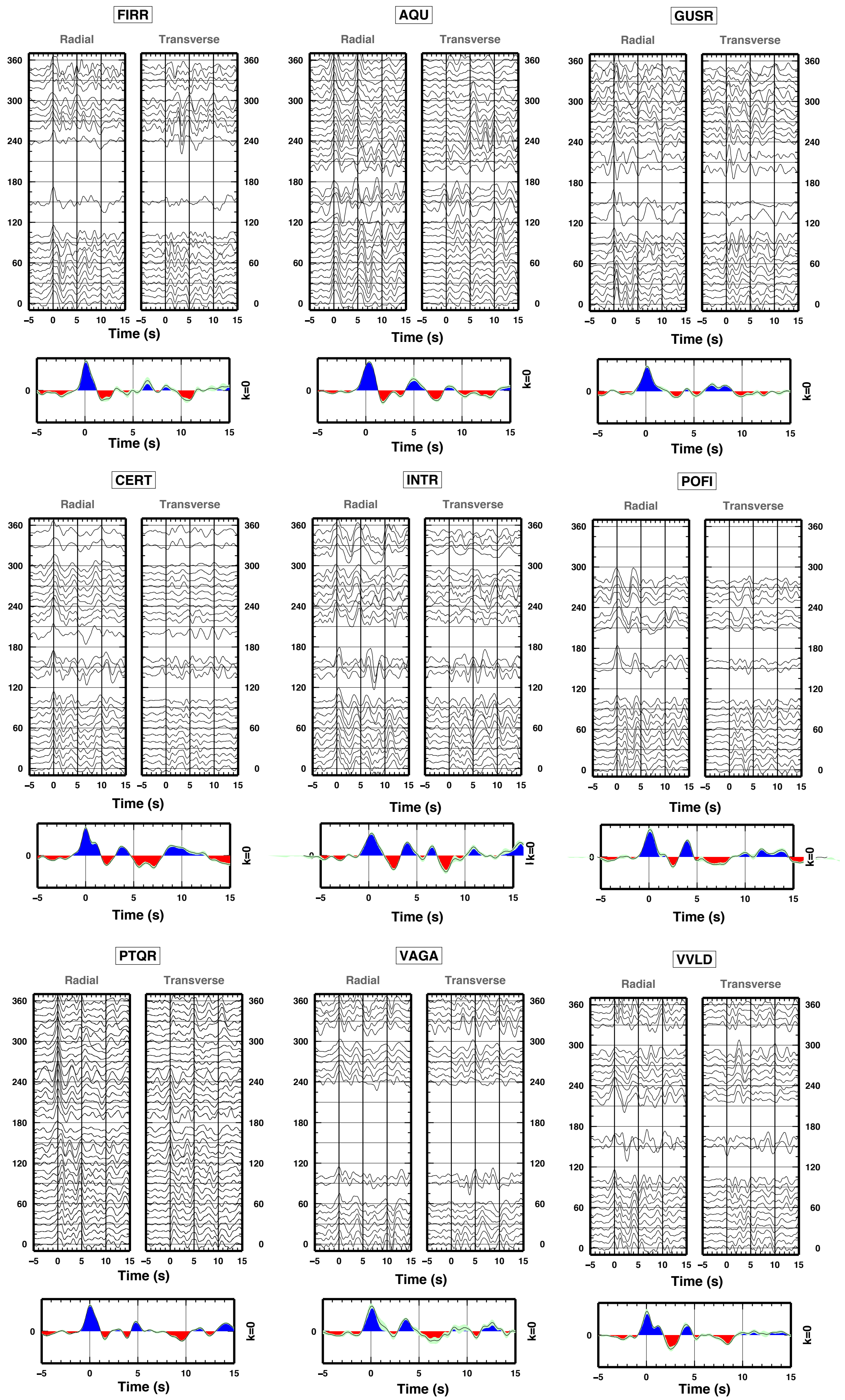
FIRR
AQU
GUSR
CERT
INTR
POFI
PTQR
VAGA
VVLD
Radial
Transverse
Time (s)
k=0
360
300
240
180
120
60
0
–5
0
5
10
15

Figure SOM2: Complete RF data-sets for some of the stations. For each station (from Northern to C-Southern Apennines) we show the radial and transverse components of the RF on the left and right panels, respectively. Numbers on the vertical axis show the back-azimuth of the incoming teleseismic events. The horizontal axis shows the time delay after the arrival of the P-direct wave. At the bottom, we show the k=0 component of the harmonics analysis as shown in Figure 3. The location of the stations is shown in Figure S1.

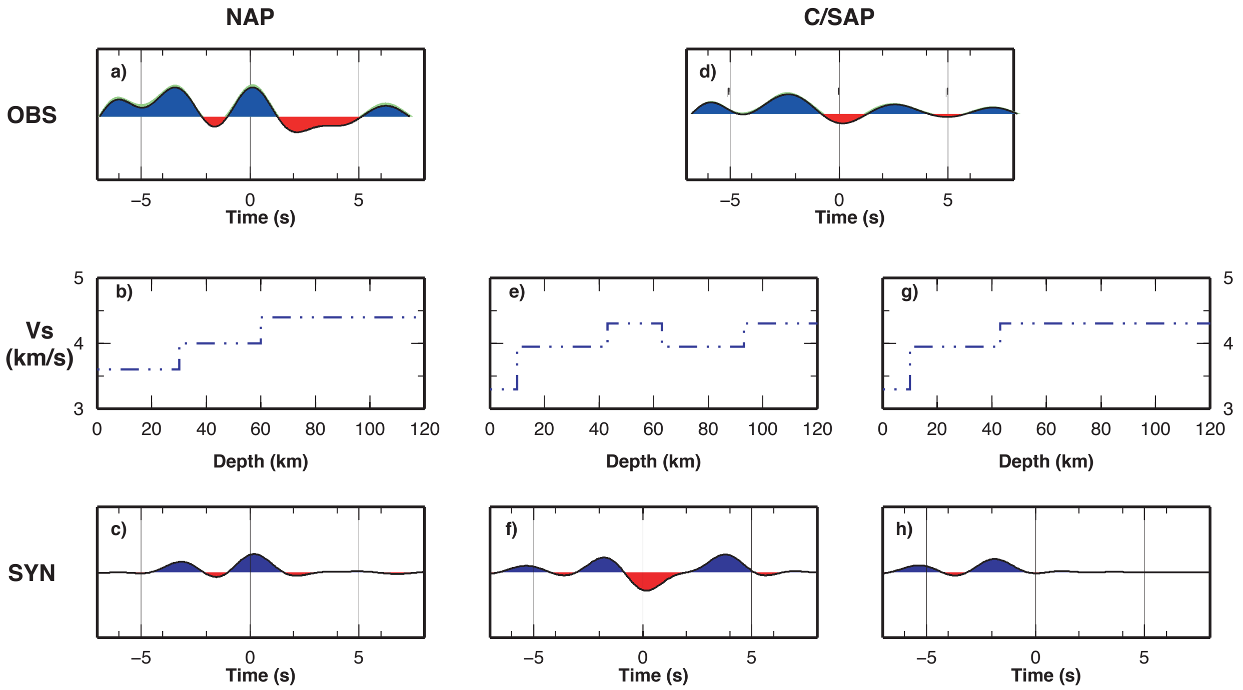

Figure SOM3: Comparison between synthetic and calculated velocity profiles for the two sectors of the Apennines. a), d) observed RF at NAP and C/SAP b), e), g) Vs models used for computing the synthetic RFs in c), f), h). In a, d, c, f, h, the RF are time shifted so that zero represents the timing of the 60 km depth.

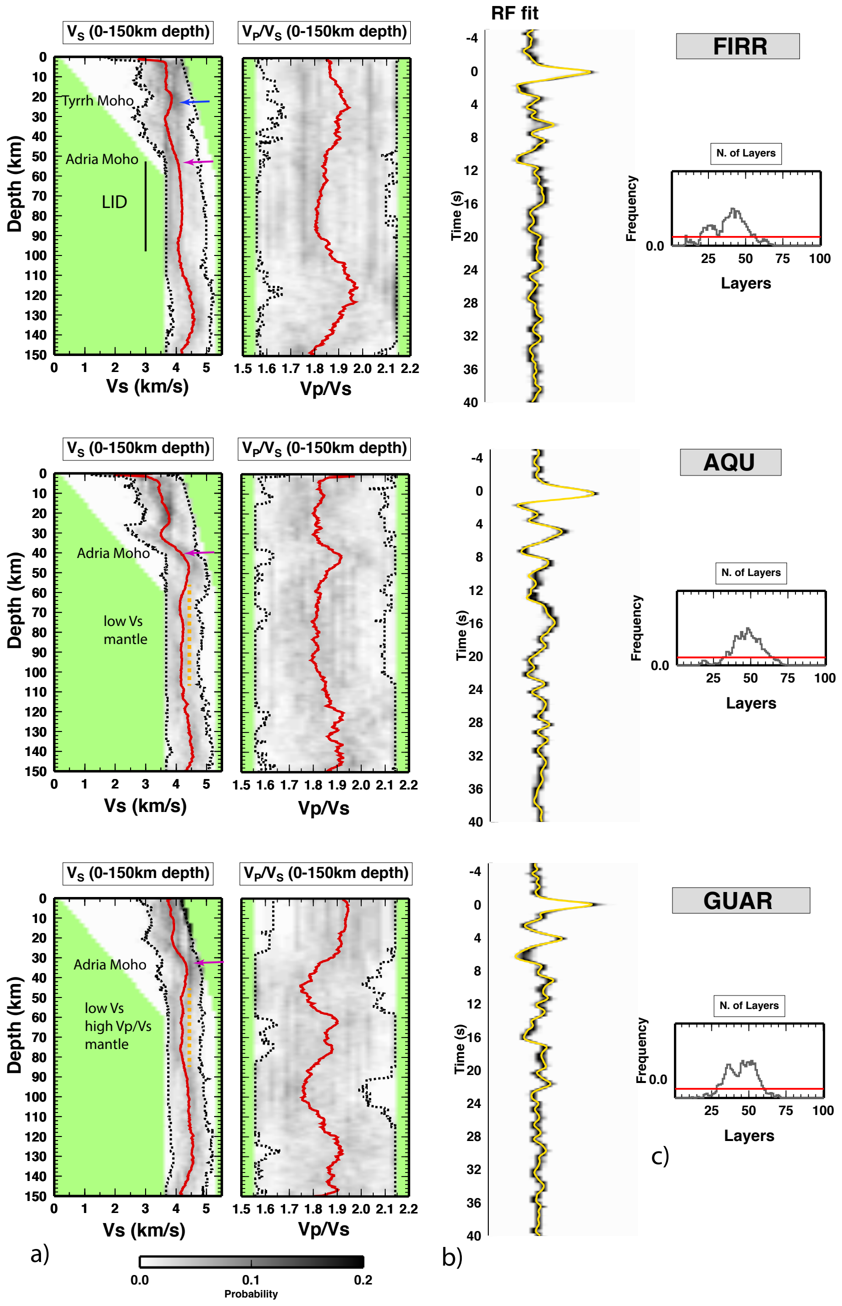

Figure SOM4: 1D velocity profiles at FIRR, AQU and GUAR representative for the NAP and C/SAP domain. a) Vs and Vp/Vs models down to 150 km depth. The Tyrrhenian and Adriatic Moho are indicated by blue and purple arrows respectively, while the orange dashed line and the orange arrows point to the low Vs and high Vp/Vs in the mantle. b) Observed (yellow) versus synthetic k = 0 harmonic of the RF. c) stations PPD of the number of layers beneath the station.

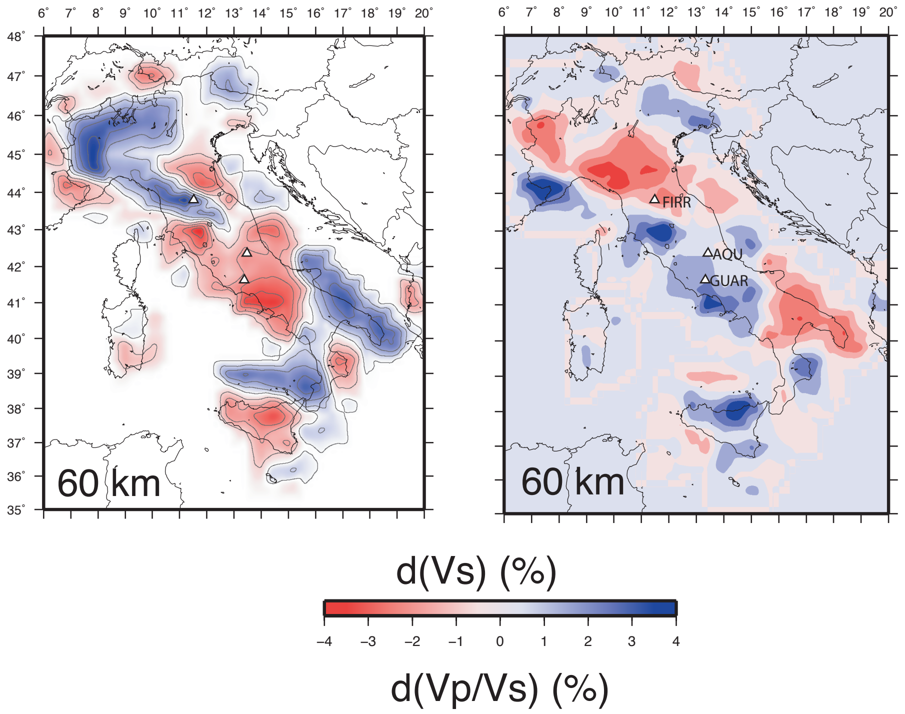

Figure SOM 5: The high resolution Vs and Vp/Vs tomographic models by Giacomuzzi et al. (2012) at 60 km depth in % of variation, informative of the structure at the top of the mantle. The model shows a broad low Vs anomaly that extends from the Tyrrhenian side to the entire low Vs area beneath the belt. The Tyrrhenian side presents a distinct high Vp/Vs, that partially extends towards the central belt. It is strikingly evident that tomographic anomalies and 1D Vs profiles for the three stations present consistent features. The three stations for which the 1D Vs profile is computed are shown by triangles.
